# 4244 A Community Partnered Research Approach to Promote Health Equity in Diverse Families

**DOI:** 10.1017/cts.2020.247

**Published:** 2020-07-29

**Authors:** Minerva Orellana, Linsey Jackson, Numra Bajwa, Melody Ouk, Norman Harrington, Melvin Anderson, Joyce Balls-Berry

**Affiliations:** 1 Mayo Clinic Graduate School of Biomedical Science

## Abstract

OBJECTIVES/GOALS: Youth and Families Determined to Succeed (YFDS), a non-profit organization in Hennepin County, MN, provides programs to address health disparities and increase health equity in diverse families. The objective of this capacity building community engaged research study was to identify factors and opportunities to expand YFDS. METHODS/STUDY POPULATION: A community partnered participatory research framework using 3 community engaged (CE) studies was conducted. This structured research process involves a facilitated discussion with a presentation on YFDS programming and a guided discussion with YFDS stakeholders. The theoretical foundation included constructs from the Model of Improvement and Health Belief Model. A trained qualitative research team led the discussion, took detailed notes, and used traditional content analysis to thematically code the notes (n = 29 pages). The studios were not audio recorded for confidentiality. Preliminary findings were presented to YFDS leadership with plans to present the results to YFDS stakeholders and families. RESULTS/ANTICIPATED RESULTS: A total of 16 YFDS past and current members participated in the studios. The average age was 42.5 years with 69% female and 75% black participants. The main themes were YFDS programming, outreach, and partnership. Participants mentioned YFDS youth “gained confidence”, found an additional family, and suggested ways to increase outreach and partnerships. Participants suggested YFDS increase their social media presence, create multicultural programing, partner with faith based organizations and schools, and determine new ways to evaluate health, social, and athletic gains. DISCUSSION/SIGNIFICANCE OF IMPACT: YFDS has positively impacted the lives of their families. With the use of CE studios, we have the opportunity to hear the voices of the members impacted that is necessary for capacity building community engaged research. We were able to find factors that made YFDS successful and suggestions to better improve and to increase positive wellness gains.

